# Prevalence, regional patterns and socio-demographic factors associated with poly-tobacco use in India: A secondary data analysis

**DOI:** 10.1371/journal.pgph.0002999

**Published:** 2024-03-15

**Authors:** Priyanka Bantwal, Muralidhar M. Kulkarni, Veena G. Kamath, Ashwath K. Naik, Andrew W. Fogarty, Murali Dhar, Anand S. Ahankari

**Affiliations:** 1 Department of Community Medicine, Kasturba Medical College, Manipal Academy of Higher Education, Manipal, Karnataka, India; 2 School of Medicine, University of Nottingham, Nottingham, United Kingdom; 3 Department of Biostatistics and Epidemiology, International Institute for Population Sciences, Mumbai, India; 4 School of Health Sciences, Faculty of Health and Medical Sciences, University of Surrey, Guildford, United Kingdom; Babcock University, NIGERIA

## Abstract

**Background:**

Tobacco use is associated with early, intermediate and long-term complications throughout the life course. With an influx of newer products containing nicotine, poly-tobacco use is slowly emerging as a public health concern, that is defined as existing tobacco users currently using two or more tobacco or nicotine products. While many studies have investigated single use tobacco, there is a paucity of research on regional patterns and socio-demographic factors associated with poly-tobacco use in India.

**Objectives:**

To assess prevalence of poly-tobacco use and determine the socio-demographic factors associated with poly-tobacco use in India.

**Methods:**

Data from the Global Adult Tobacco Survey 2 (GATS, 2016–17) was analysed, which included information on tobacco use among people aged >15 years. The pattern of current tobacco status was described using descriptive statistics. Multiple logistic regression models were estimated to determine factors associated with poly-tobacco use.

**Results:**

The prevalence of poly-tobacco use in India was found to be 9.8%. Among the current tobacco users, the prevalence was 33%. Significant socio-demographic factors associated with poly-tobacco use included younger age, male gender, religion and backward caste. North-eastern region reported highest prevalence of poly-tobacco use in the country, followed by the central region.

**Conclusion:**

The number of poly-tobacco users in India is considerably high and a matter of concern, more so in north east and central regions of the country. There is a need to create awareness about dangerous effects of all types of tobacco products and strengthen implementation of tobacco control policies with special focus on regions with high burden.

## Introduction

Tobacco use is a leading preventable cause of death in the world [1, 2]. It is a causative factor for cancers, ischemic heart disease, cerebro-vascular disease, lower respiratory infections, tuberculosis and chronic obstructive pulmonary disease [3]. Tobacco consumption has a strong impact on the global economy with smoking costing about US$1436 billion, which is roughly 1.8% of yearly global Gross Domestic Product (GDP) [4]. Nearly 40% of this is borne by the low- and middle-income countries (LMICs), demonstrating the significant damage that tobacco use causes in LMICs [4]. Similar trends are seen in India where economic costs caused due to tobacco use accounts for 1.04% of India’s GDP [5]. Total economic costs attributable to tobacco use from all diseases in India in the year 2011 for persons aged 35–69 years amounted to US$ 22.4 billion, of which 16% was direct and 84% was indirect cost [6]. Furthermore, the use of tobacco increases the likelihood of poverty by diverting household funds away from essentials like food and family necessities [7], which is a concern in LMICs.

Recognizing the multi-level threat that tobacco holds for the future, the World Health Organisation (WHO) negotiated the first global treaty: Framework Convention on Tobacco Control (FCTC) that brought different countries together to achieve the common goals of actively working towards tobacco control, while ensuring healthy living and wellbeing for all [8]. India ratified this treaty and subsequently introduced the Cigarettes and Other Tobacco Products Act (COTPA) in 2003 that regulates and controls the manufacturing, distribution, and sales of various tobacco products [9]. Later in 2007–2008, the National Tobacco Control Programme (NTCP) was launched, which aims to educate people about the dangers of tobacco, efficiently administer COTPA laws and strengthen cessation facilities [10].

With consistent efforts from countries across the globe, the prevalence of tobacco use has declined in majority of the countries over the years [11]. However, newer forms of tobacco products like cigars, e-cigarettes, vaping pens and hukkah are on a rise [12, 13], thus leading to poly-tobacco use. This has been defined as consumption of multiple tobacco or nicotine products [14, 15] and is proving to be a serious threat to the public health [15, 16]. A systematic review on nationally representative data from 48 countries on adult population has estimated that the poly-tobacco use is increasing in South-East Asia [17].

Studies have reported that the concurrent use of multiple tobacco products makes people vulnerable to nicotine dependence, addiction and substance use disorders [18, 19]. Interplay of factors like personal circumstances (e.g., socioeconomic status, peer susceptibility, nicotine dependence), situational factors (e.g., peer use, product susceptibility) and person-product interaction (e.g., reinforcement, product knowledge, perceived harm) explain the initiation and continuation of multiple tobacco products among people [20]. Similar trends have been seen with alternative tobacco products like e-cigarettes and vaping pens [21–24]. These are often marketed as safer alternatives to the traditional products like cigarettes and smokeless tobacco products, that causes less harm [25, 26] and thus, aid in cessation. However, studies have found that they contain chemicals and compounds that can cause equal damage to the health of the person, as with traditional tobacco products [27, 28]. Despite a blanket ban on sales of e-cigarettes in India [29], people are still able to access different tobacco products [30], leading to its continued use [31, 32]. Thus, availability of a range of tobacco products has led users to experiment various options.

Use of tobacco itself causes a spectrum of difficulties for people. With easy availability of newer variants of tobacco products in the market, along with the emergence of a greater number of poly-tobacco users [14, 33], it is pertinent to understand patterns, prevalences and factors associated with such users to develop strategies to better manage the emerging crisis of poly-tobacco users in India. Several research papers have been published using the Global Adult Tobacco Survey 2 (GATS2) database, however to our knowledge, the component of poly-tobacco use has not been explored. Therefore, this secondary data analysis was conducted to investigate the pattern, prevalence and factors associated with poly-tobacco use in India.

## Methods

### Data source

The analysis was based on data from the second round of GATS 2 survey conducted in India during 2016–2017 by the Ministry of Health & Family Welfare, that is available in the public domain for research and education purposes [34]. It provides information on tobacco use behaviour among the adult population (above 15 years of age) living in their primary residence along with tracking key tobacco control indicators such as cessation, second hand smoke, economics, industry advertisement and anti-tobacco information [35]. Demographic characteristics included age, residence, sex, religion, caste, marital status, level of education, and working status.

The GATS gathered data on the consumption of various smoking tobacco products including manufactured cigarettes, rolled tobacco in paper or leaf, bidis, cigars, cheroots, or cigarillos, hukkah and any other smoking products. The smokeless tobacco products included betel quid, khaini or tobacco lime mixture, gutka, areca nut-tobacco lime mixture, or mawa, oral tobacco use (as mishri, qul, gudakhu), nasal use of snuff, and any other smokeless products [36]. In addition, information about the use of other nicotine-based products such as e-cigarettes were also incorporated in the questionnaire. Further details on data collection procedures and data collected through GATS 2 are available online [37]. Responses to the following GATS question was used to categorize respondents as current cigarette smoker/non-smoker: *‘On an average*, *how many of the following products do you currently smoke each day*? *Also*, *let me know if you smoke the product*, *but not every day’*. Those who reported smoking one or more cigarettes each day, or smoking cigarettes but not every day, were considered as current cigarette smokers; whilst those who indicated that they did not smoke any cigarette were classed as non-smokers. The current users of other tobacco product users were also determined in a similar way. For data on water pipe and e-cigarettes products, the following question was used: *Do you currently smoke on a daily basis*, *less than daily*, *or not at all*? Those who responded to their use status to be “daily” or “less than daily” were considered users of e-cigarettes and water pipe, while those who answered as “not at all” were grouped as non-users. When responses were blank or the respondent declined to answer, values were treated as missing. Observations with missing values for any of the variables pertaining to tobacco use were excluded from our data analysis.

### Ethics statement

This is a secondary data analysis of a database available in the public domain. This data is de-linked, anonymised and cannot be traced back to individual participants. Thus, there was no violation of confidentiality of the participants.

### Operational definition

Poly-tobacco users are defined as existing tobacco users currently using two or more tobacco or nicotine products. In contrast, exclusive “single” tobacco product users are those who currently reported using only one tobacco product.

### Statistical analysis

The analysis was performed using SPSS software for Windows (SPSS22.0, IBM, USA). By using sociodemographic information gathered from the GATS 2, descriptive analysis summarising the current tobacco users at the time of the survey and the proportions of poly-tobacco users was conducted. Participants were grouped into quintiles of family wealth (poorest, poor, middle, wealthier, or wealthiest) based on various household possessions [38]. After estimating the prevalence of poly-tobacco use among current users, binary logistic regression analysis was used to establish a risk model to assess the role of potential explanatory variables. Socio demographic variables that were found to be significantly associated with poly-tobacco use on bivariate analysis were evaluated by multiple logistic regression analysis. The odds ratios (ORs) and 95% confidence interval (CIs) between the covariate and outcome variables were calculated and used as the measures of association. We considered estimates to be statistically significant if the p-value from a two-tailed test was less than 0.05.

## Results

**Fig 1:**
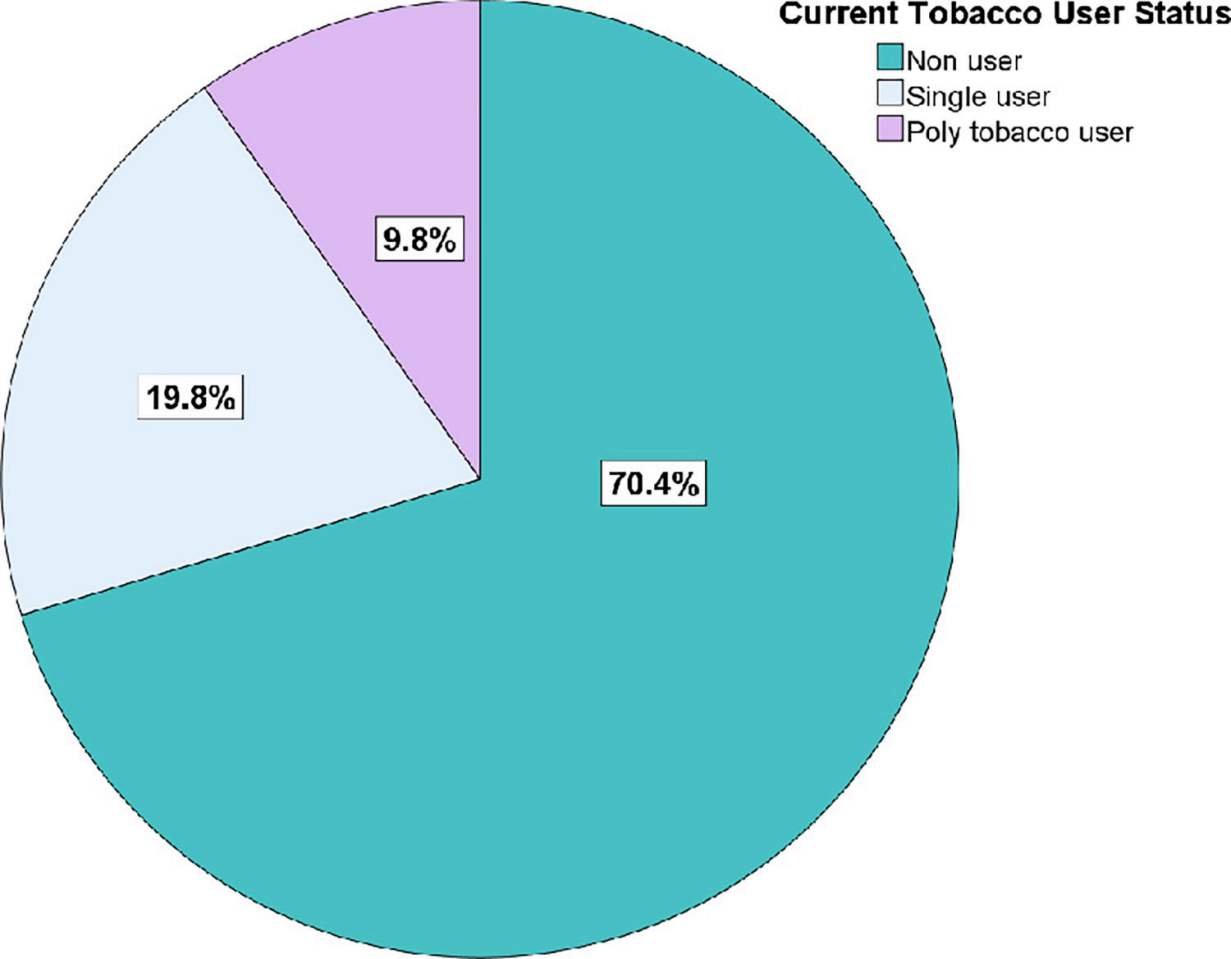
Prevalence of current single and poly-tobacco users among people aged 15 years and above, India (2016–17).

GATS-2 Survey data was collected on 74,037 individuals, from which 99.9% of the observations (73998/74037) was used for analysis after excluding 39 observations with missing values for current tobacco use. As indicated in the **Fig 1**, 70.4% (52103/73998) of GATS 2 respondents were non-users, 19.8% (14669/73998) were single tobacco users, 9.4% (6942/73998) users consumed 2 to 5 tobacco products, and 0.4% (284 / 73998) users consumed more than six tobacco products. The tobacco use prevalence was 29.6% (21895/73998), with 67% (14669/ 21895) of these being single product users and 33% (7226 / 21895) were poly-tobacco users.

Among the tobacco products, “khaini or tobacco lime mixture” (10.1%) was the most commonly used, followed by “betel quid with tobacco” (6.8%), “Gutka” (5.1%), “Oral tobacco use” (3.8%), “Pan masala together with tobacco” (2.5%) and “nasal use of snuff” (0.5%) in smokeless tobacco products. In smoking tobacco products “rolled tobacco in paper or leaf” (8.2%) was used more frequently, followed by “cigarettes” (4.5%), “bidis” (1.8%), “hukkah” (0.9%) and “cigars” (0.4%). Description of current tobacco prevalence for all products is detailed in the **Table 1**.

**Table 1:** Number (n) and proportion (%) of current tobacco user based on type of products used among people aged 15 years and above, India (2016–17).

Tobacco types	Products	Current Users n (%)
Smoking	Manufactured cigarettes	3339 (4.5%)
	Bidis	1297 (1.8%)
	Rolled tobacco in paper or leaf	6070 (8.2%)
	Cigars, cheroots, or cigarillos	329 (0.4%)
	Number of hukkah sessions	699 (0.9%)
	Any others form of smoking tobacco	203 (0.3%)
Smokeless	Betel quid with tobacco	5054 (6.8%)
	Khaini or tobacco lime mixture	7476 (10.1%)
	Gutka, areca nut—tobacco lime mixture, or mawa	3803 (5.1%)
	Oral tobacco use (as mishri, gul, gudakhu)	2787 (3.8%)
	Pan masala together with tobacco	1875 (2.5%)
	Nasal use of snuff	397 (0.5%)
	Any other form of smokeless tobacco	318 (0.4%)
Water pipe	Water pipe	157 (0.2%)
e-cigarettes	e-cigarettes	32 (0.04%)

**Fig 2:**
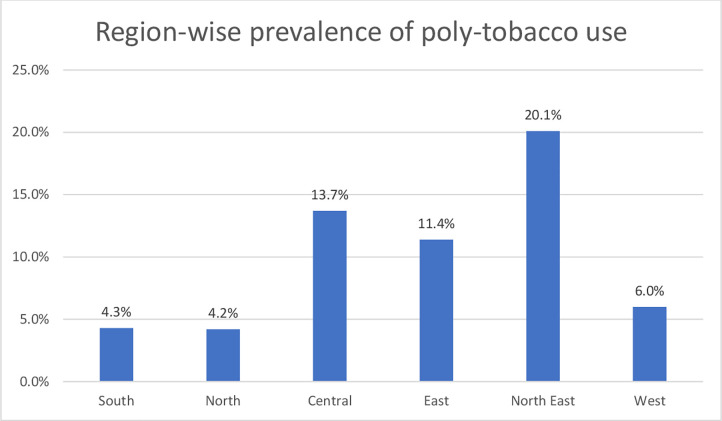
Region—wise prevalence of poly-tobacco use aged 15 years and above, India (2016–17).

**Fig 2** illustrates region wise analysis of poly-tobacco use in India. These results show that north-east region had the highest prevalence (20.1%), followed by central region (13.7%) for poly-tobacco use.

**Table 2** presents results from the binary logistic regression exploring associations between socio demographic factors and poly-tobacco use. In univariate analysis the prevalence of poly-tobacco use was significantly associated with younger age groups and was more likely among men, those who were not Hindus, among the schedule tribal (ST) communities and other backward class (OBC) population, those who were single, those residing in other than South region, and people with lower education levels. All factors included in the model reported significant association in univariate analysis (p<0.05) and thus all were included in the multivariate model. Prevalence of poly-tobacco use was higher in the age group of 25 to 44 years compared to those aged 65 years and above (aOR = 1.5, 95% CI: 1.4 to 1.7). In comparison to females, males had four times greater odds of being poly-tobacco users (aOR = 1.6, 95% CI: 1.5 to 1.8). Christian (aOR = 1.3, 95% CI: 1.2 to 1.5) and other religions (aOR = 1.4, 95% CI: 1.2 to 1.6) were more likely to be poly-tobacco users than Hindus. Compared to those in Schedule caste, those from other backward classes were more likely to be poly-tobacco users (aOR = 1.1, 95% CI: 1.0 to 1.2). Compared to those residing in the South, all other regions had higher proportions of poly-tobacco users **(Fig 2)**. Furthermore, Central and North-East regions had highest odds of having poly-tobacco users compared to other regions **(Table 2)**. There was no significant association between prevalence of poly-tobacco use and marital status, education or working status.

**Table 2:** Prevalence of poly-tobacco use and factors associated with poly-tobacco users among current tobacco users (univariate and multivariate analysis), India 2016–17.

Variable	Total Current Tobacco Users (n = 21895)	Poly tobacco users (n = 7226) n (%)	Crude OR* (95% CI)	p-value	Adjusted OR (95% CI)	p-value
**Age group**				**<0.001**		**<0.001**
≥65	2243	543 (24.2%)	1		1	
15–24	1848	696 (37.7%)	1.9 (1.7,2.2)		1.5 (1.2,1.7)	
25–44	10845	3904 (36.0%)	1.8 (1.6,2.0)		1.5 (1.4,1.7)	
45–64	6959	2083 (29.9%)	1.3 (1.2,1.5)		1.3 (1.1,1.4)	
**Residence**				0.194		
Rural	16407	5454 (33.2%)	1			
Urban	5488	1772 (32.3%)	1.0 (0.9,1.0)			
**Gender**				**<0.001**		**<0.001**
Female	6279	1691 (26.9%)	1		1	
Male	15616	5535 (35.4%)	1.5 (1.4,1.6)		1.6 (1.5,1.8)	
**Religion**				**<0.001**		**<0.001**
Hindu	15230	4755 (31.2%)	1		1	
Muslim	2695	848 (31.5%)	1.0 (0.9,1.1)		1.0 (0.9,1.1)	
Christian	2995	1239 (41.4%)	1.6 (1.4,1.7)		1.3 (1.2,1.5)	
Others	958	378 (39.5%)	1.4 (1.3,1.6)		1.4 (1.2,1.6)	
**Caste**				**<0.001**		**0.002**
SC	4060	1262 (31.1%)	1		1	
ST	5750	2144 (37.3%)	1.3 (1.2,1.4)		0.9 (0.8,1.0)	
OBC	7044	2329 (33.1%)	1.1 (1.0,1.2)		1.1 (1.0,1.2)	
Others	4869	1411 (29.0%)	0.9 (0.8,1.0)		0.9 (0.8,1.0)	
**Marital status**				**<0.001**		0.085
Married	18253	6003 (32.9%)	1		1	
Single	2013	795 (39.5%)	1.3 (1.2,1.5)		1.1 (1.0,1.3)	
Separated, Divorced and widowed	1627	427 (26.2%)	0.7 (0.7,0.8)		1.1 (1.0,1.2)	
**Region**				**<0.001**		**<0.001**
South	2562	610 (23.8%)	1		1	
North	2848	725 (25.5%)	1.1 (1.0,1.2)		1.1 (0.9,1.2)	
Central	4145	1573 (37.9%)	2.0 (1.8,2.2)		2.0 (1.8,2.2)	
East	3655	1120 (30.6%)	1.4 (1.3, 1.6)		1.5 (1.3,1.7)	
North East	6922	2728 (39.4%)	2.1 (1.9, 2.3)		2.0 (1.8,2.3)	
West	1763	470 (26.7%)	1.2 (1.0,1.3)		1.2 (1.0, 1.4)	
**Education status**				**<0.001**		0.080
Graduation and above	1083	351 (32.4%)	1		1	
No formula education	6882	2049 (29.8%)	0.9 (0.8,1.0)		1.3 (1.1,1.5)	
Primary incomplete	3214	1091 (33.9%)	1.1 (0.9,1.2)		1.2 (1.0,1.5)	
Primary complete but not secondary completed	7022	2467 (35.1%)	1.1 (1.0,1.3)		1.2 (1.0,1.4)	
Secondary and higher education complete	3681	1261 (34.3%)	1.1 (0.9,1.3)		1.2 (1.0,1.3)	
**Work status**				**<0.001**		0.219
Government and non–government employee	2707	942 (34.8%)	1		1	
Daily wage and casual labour	6962	2398 (34.4%)	1.0 (0.9,1.1)		1.0 (0.9,1.1)	
Self employed	6417	2245 (35.0%)	1.0 (0.9,1.1)		1.0 (0.9,1.1)	
Student	425	160 (37.6%)	1.1 (0.9,1.4)		0.9 (0.7,1.1)	
Home maker	3627	947 (26.1%)	0.7 (0.6,0.7)		0.9 (0.8,1.0)	
Unemployed	1216	413 (34.0%)	1.0 (0.8,1.1)		1.0 (0.9,1.2)	
**Wealth quintile**				0.108		0.751
Upper	2038	669 (32.8%)	1		1	
Lower	6649	2248 (33.8%)	1.1 (0.9,1.2)		1.0 (0.9, 1.1)	
Lower–Middle	4710	1562 (33.2%)	1.0 (0.9,1.1)		1.0(0.8,1.1)	
Middle	5351	1689 (31.6%)	0.9 (0.9, 1.1)		1.0 (0.9,1.1)	
Middle—Upper	3147	1058 (33.6%)	1.0 (0.9,1.2)		1.0 (0.9,1.2)	

* p<0.1 after adjustment for age, gender, religion, cast, marital status, region, education status, work status and wealth quintile.

## Discussion

The current study aimed to understand pattern and prevalence of poly-tobacco users in India and determine socio-demographic factors associated with poly-tobacco use by conducting secondary analysis of the GATS 2 database. The overall prevalence of poly-tobacco in India was 9.8%. In this nation-wide survey (having 74,037 people aged 15 years and above), 28.6% were current tobacco users that included smoking and smokeless products. The survey also included use of tobacco products like water pipe and e-cigarettes that results in prevalence of current tobacco use to be 29.6%. The proportion of poly-tobacco use reported among current tobacco users was 33%. Our analysis showed that north-east and central regions of India had the highest burden of poly-tobacco use, with similar trends seen in single tobacco use as well. The significant factors associated with poly-tobacco use were younger age groups, male gender, other religion, backward caste and being in the north east region.

The prevalence of poly-tobacco use has increased to 9.8% in GATS 2 survey from 6.5% as reported in the GATS 1 (2009–10) [33]. The prevalence of poly-tobacco use among South-East Asian countries, is similar in Bangladesh (8.8%), followed by Thailand (5.9%). Globally, Denmark (11.9%) and United Kingdon (11.4%) reported highest poly-tobacco use [14]. This suggests that poly-tobacco use is a concern not only in India but across the globe, which needs attention.

Among the current tobacco users, poly-tobacco use was high (33%), which is a substantial increase from 21.5% as reported in a study based on GATS 1 data [39]. This considerable increase could be due to various reasons. A study done in Bangladesh analysed data from 2009 to 2015 and found social factors like friends, family along with false beliefs regarding various tobacco products significantly influenced the decision to use multiple products [40]. Studies have also highlighted exposure to advertisements of tobacco products as a key role leading to consumption of a variety of tobacco products [21]. India has a range of tobacco products and current evidence suggests that poly-tobacco use is one of the major categories of tobacco use. Studies have found large differences in the prices of various smokeless [41] as well as smoking [42] tobacco products in Asian region, which results in tobacco users switching to lower priced products. Thus, even with ongoing inflation, they are able to have access to other affordable tobacco products thereby leading to poly-tobacco use [43, 44]. Companies use flavours to attract customers, which in turn plays a role in use of multiple tobacco products [22, 45]. Continuous exposure to nicotine [46] and means to cope with low mood [47] could be some of the reasons for accessing diverse range of tobacco products, leading to long term dependency on nicotine.

Results from our study highlighted that age and gender were significant factors that determined poly-tobacco use in India. The results were supported by a recent review that analysed cross-country data on tobacco from the Demographic and Health Survey (DHS) among 19 LMICs [48]. Similar results were noted among American population through their Population Assessment of Tobacco and Health Study (PATH: 2013–14) which found significant association among younger adults (12–17 years) and male gender for poly-tobacco use [49].

Our study also found that the prevalence of poly-tobacco use was the highest among people in the north-eastern and central regions. Our results are supported by data on state wise prevalence of tobacco use in India, which indicates high prevalence of tobacco in states from these regions, that is much greater than the national average [50–52]. Studies have also reported greater use among students, women and tribal population in this region [53–56]. There have been studies that have highlighted that wealth quintile is an important factor that affects tobacco use [57, 58]. A study based on the National Sample Survey (NSS) analysed trends in socio-economic inequalities in tobacco in India from 2000 to 2012 found a 17% increase in tobacco use among Scheduled Tribe over the years, whereas in the present study backward caste was a significant determinant for poly-tobacco use [59].

### Conclusions

Tobacco use threatens the public health, economic growth and development of the country. Despite reduction in prevalence of tobacco use over the years, there has been a steady increase in the number of poly-tobacco users in the country. Our study demonstrates the need to continue persistent efforts through tobacco control, while also responding to new challenges of emerging tobacco products. The results of our study indicate the need for stronger enforcements of the tobacco control policies across all age groups and regions of the country to reduce tobacco consumption from a range of products, ensure implementation of the national health policy and achieve tobacco related Sustainable Development Goals in the country. Tobacco control strategies should consider the local patterns of consumption and be tailored to individual needs, which may vary depending on the products involved. We recommend awareness campaigns that highlight negative health effects of all types of tobacco products that would avoid myths of safer tobacco products.
